# Interstitial Lung Disease and Psoriasis in a Child With Aicardi-Goutières Syndrome

**DOI:** 10.3389/fimmu.2020.00985

**Published:** 2020-05-20

**Authors:** Shaoling Zheng, Pui Y. Lee, Jun Wang, Shihao Wang, Qidang Huang, Yukai Huang, Yuqi Liu, Qing Zhou, Tianwang Li

**Affiliations:** ^1^Department of Rheumatology and Immunology, Guangdong Second Provincial General Hospital, Guangzhou, China; ^2^Division of Immunology, Boston Children's Hospital, Harvard Medical School, Boston, MA, United States; ^3^Life Sciences Institute, Zhejiang University, Hangzhou, China

**Keywords:** Aicardi-Goutières syndrome, IFIH1/MDA5, interstitial lung disease, psoriasis, pulmonary hypertension

## Abstract

Aicardi-Goutières syndrome (AGS) is characterized by progressive neurologic decline, cerebral calcification, and variable manifestations of autoimmunity. Seven subtypes of AGS have been defined and aberrant activation of the type I interferon system is a common theme among these conditions. We describe a 13-year-old boy who presented with an unusual constellation of psoriasis, interstitial lung disease (ILD), and pulmonary hypertension in addition to cerebral calcifications and glomerulonephritis. He was found to have late-onset AGS due to a gain-of-function mutation in *IFIH1* and over-activation of the type I interferon pathway was confirmed by RNA sequencing. The majority of his clinical manifestations, including ILD, psoriasis and renal disease improved markedly after treatment with the combination of corticosteroids, cyclophosphamide, and the Janus-kinase inhibitor tofacitinib. This case extends the clinical spectrum of AGS and suggests the need for lung disease screening in patients with AGS.

## Introduction

Aicardi-Goutières syndrome (AGS) is a group of inherited disorders characterized by early-onset neurologic decline and autoimmunity (1). Seven types of AGS have been identified based on the causative genes: *TREX1, RNASEH2A, RNASEH2B, RNASEH2C, SAMHD1, ADAR1*, and *IFIH1* (2). These genes normally function to modify nucleic acids or sense cytoplasmic nucleic acids. AGS-associated mutations typically result in aberrant production of type I interferons, a class of cytokines involved in anti-viral defense. This mechanism positions AGS among the interferonopathy family of autoinflammatory disorders (3).

Primary manifestations of AGS include progressive developmental decline, encephalopathy, cerebral calcification, muscle weakness, or spasticity, and variable features of autoimmunity ranging from the presence of autoantibodies to full-blown presentation of systemic lupus erythematosus (SLE) (2). Chilblain-like rash in the extremities exacerbated by cold temperature is the most common skin involvement (4). Parenchymal lung disease is uncommon although pulmonary hypertension has been described (5).

Here we present a case of late-onset AGS with a gain-of-function mutation in *IFIH1* (6, 7). Expanding the clinical spectrum of AGS, we describe unusual features of interstitial lung disease (ILD) and psoriasis in addition to neurologic and immunologic abnormalities in this patient.

## Case Presentation

**Figure 1:**
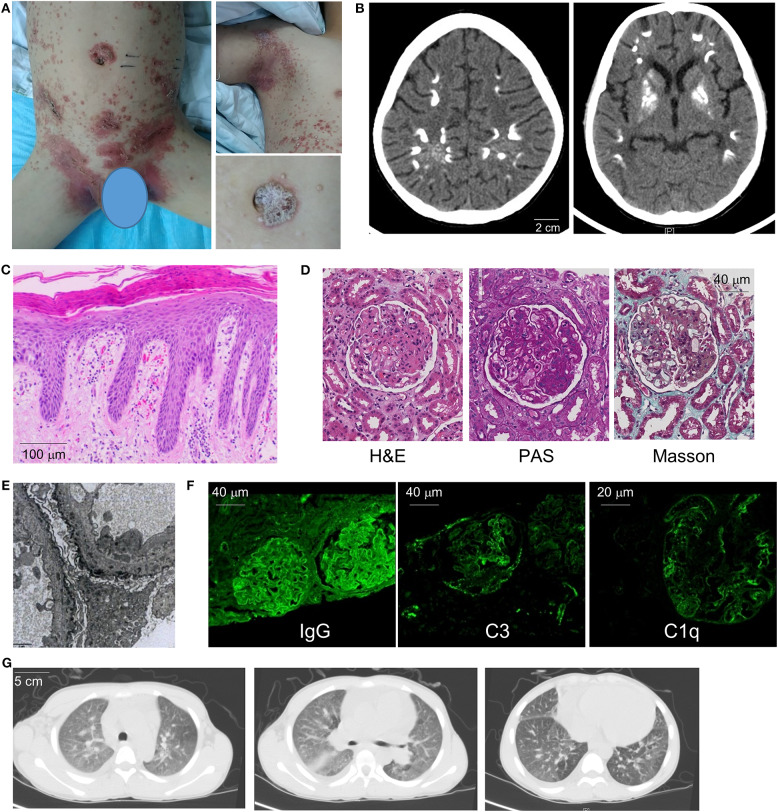
Clinical manifestations and diagnostic evaluation. **(A)** Images of the patient's generalized psoriatic rash in the trunk (left) and axilla (upper right) at the time of initial presentation. Magnified panel (lower right) illustrates features of plaque psoriasis. **(B)** Brain CT demonstrates areas of calcification in the cerebral cortex and basal ganglia. **(C)** Skin biopsy illustrates hallmark features of psoriasis including hyperkeratosis, dilated capillary loops with a perivascular lymphocytic infiltrate, mild dermal edema, and regular psoriasiform epidermal hyperplasia with elongation of the rete ridges. **(D)** Renal biopsy pathology (H&E, PAS, and Masson's stains) illustrates membranous nephropathy and mesangial hyperplasia. **(E)** Electron microscopy of renal tissue demonstrates electron-dense deposits along the basement membrane (8000× magnification). **(F)** Immunofluorescence of renal tissue reveals positive glomerular staining for IgG, complement C3 and C1Q. **(G)** Chest CT images illustrate small pleural effusion, diffuse ground-glass opacities, and emphysema of lower lobes.

We present a 13-year-old boy who developed a psoriasis-like rash and progressive weakness of lower extremities at the age of 11. The rash initially involved the scalp but gradually expanded to affect the ears, armpit, back, abdomen, scrotum, and lower extremities (Figure 1A). The Physician's Global Assessment of Psoriasis (PGA-PsO) score was 3, indicative of moderate disease severity. Soon after the onset of skin rash, he developed lower extremity weakness that progressed to inability to ambulate independently. Brain computerized tomography (CT) scan showed multiple calcifications in the cerebral cortex and basal ganglia (Figure 1B), which led to a diagnosis of Fahr's syndrome at a local hospital.

The patient was transferred to our hospital due to progressive muscle weakness. On admission, his physical exam was notable for the diffuse psoriasiform rash with areas of pustulosis, shortness of breath with exertion, severe weakness of lower extremities (3-/5 vs. 5/5 for upper extremities), and clonus upon ankle flexion. Lungs were clear to auscultation. No evidence of joint inflammation or deformity was noted on musculoskeletal exam.

Initial laboratory investigations revealed normal complete blood count, low albumin, low complements (C3 and C4) and severe proteinuria (Table 1). Immunologic studies showed positive anti-nuclear antibodies (1:100), positive PR3-ANCA (proteinase 3-specific anti-neutrophil cytoplasmic antibodies), and elevated levels of serum cytokines including IL-6, IL-8, and IL-1β. The patient also exhibited features of autoimmune thyroid disease with autoantibodies and impaired thyroid function (Table 1).

**Table 1:** Laboratory data before and after treatment.

	**Reference**	**Pre-treatment**	**Post-treatment (3 Mo)**
WBC (× 10^9^/L)	3.5–9.5	6.1	4.9
Hemoglobin (g/dL)	13.0–17.5	15.4	14.2
Platelet (× 10^9^/L)	125–350	247	295
Urinary protein (dipstick)	–	++++	++
Urinary protein (mg/2 4h)	0–150	3,210	1,262
Urinary protein (mg/L)	0–150	10,700	789.31
Urine creatinine (μmol/L)	–	6,857	2,763
Serum creatinine (μmol/L)	40–140	72	88
Blood urea nitrogen (mmol/L)	2.5–7.5	1.89	3.38
Triglycerides (mmol/L)	0.4–1.8	2.85	3.94
Albumin (g/L)	35–50	22.7	34.7
Creatine kinase (U/L)	26–218	39	31
Lactate dehydrogenase (U/L)	80–200	222	197
ALT (U/L)	0–50	39	25
AST (U/L)	0–50	34	19
Pro-BNP (pg/mL)	0–150	97.9	n/d
CK (U/L)	26–218	39	n/d
LDH (U/L)	222	80–240	n/d
C-reactive protein (mg/L)	0–8	3.1	0
ESR (mm/h)	0–15	30	7.8
C3 (g/L)	0.8–1.6	0.4	1.17
C4 (g/L)	0.1–0.5	0.07	0.12
IgG (mg/dL)	8.0–16	11.9	9.7
IgA (g/L)	0.7–3.3	3.34	1.02
IgE (g/L)	0–200	1,210	1,770
IL-6 (pg/ml)	0–5.4	88.7	13.7
IL-8 (pg/ml)	0–20.6	342.4	101.5
IL-1β (pg/ml)	0–12.4	48	0
TNF-α (pg/ml)	0–16.5	3.2	1.3
Free T3 (pmol/L)	3.5–6.5	1.84	5.65
Free T4 (pmol/L)	11.0–23	7.65	13.15
TSH (μIU/mL)	0.35–5.5	>100	13.08
anti-TG antibodies (U/ml)	0–60	534.7	21.26
anti-TPO antibodies (U/ml)	0–60	258.5	27.54
Parathyroid hormone (pg/ml)	15–65	69.48	40.68
Antinuclear antibodies	Negative	1:100	n/d
ANCA	Negative	+PR3-ANCA	n/d

*BNP, B-type natriuretic peptide; TSH, thyroid stimulating hormone; TG, thyroglobulin; TPO, thyroperoxidase*.

The patient underwent extensive medical evaluation given the multi-organ system disease involvement. Electromyography and bone marrow biopsy were unremarkable. Skin biopsy showed squamous epithelial hyperkeratosis, mild dermal edema, and perivascular lymphoid aggregates, consistent with features of psoriasis (Figure 1C). Renal biopsy showed drastic mesangial hyperplasia and membranous nephropathy while electronic microscopy illustrated dense deposits along the basement membrane compatible with nephritis secondary to an autoimmune process (Figures 1D,E). In line with this view, immunofluorescence showed glomerular staining for IgG(+ + +), C3(++), C1q(++), IgA(++), and IgM(++) (Figure 1F and not shown).

Cardiopulmonary evaluation was performed due to the patient's shortness of breath on exertion. Echocardiogram found cardiomegaly with small to moderate pericardial effusion but otherwise structurally normal heart without evidence of valve disease. Moderate pulmonary hypertension was noted with a systolic pulmonary arterial (PA) pressure of 48 mmHg. CT chest showed evidence of interstitial lung disease with diffuse ground glass opacities and emphysema of the lower lobes. Enlarged lymph nodes adjacent to the pulmonary hilum and mild pleural effusion were also noted (Figure 1G). Pulmonary function test showed moderate restrictive lung disease and severe diffusion defect (FEV1: 67% predicted; FVC: 64% predicted; DLCO: 33% predicted).

**Figure 2:**
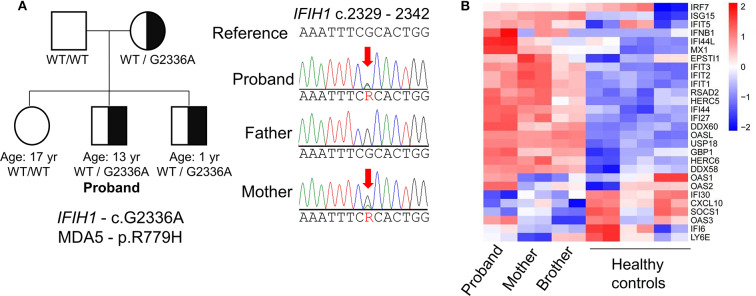
Genetic evaluation of Aicardi-Goutières syndrome. **(A)** Identification of a missense mutation in *IFIH1* by whole-exome sequencing and pedigree of the patient's family. The proband shares the pathogenic mutation with his mother and younger brother. **(B)** Heat-map of RNA sequencing analysis illustrates upregulation of IFN-I-inducible genes in the proband compared to healthy controls. The mother and brother display a milder interferon signature compared to the proband.

Given the unusual combination of systemic features, whole exome sequencing (WES) was sent and the patient possessed a pathogenic variant in *IFIH1* (c.G2336A:p.R779H; Figure 2A), which encodes melanoma differentiation-associated protein 5 (MDA5). This gain-of-function variant located in helicase domain 2 of MDA5 was previously identified in patients with AGS (7, 8). The patient's mother was found to have the same mutation but she is healthy without any medical concerns. Subsequent serologic testing for the mother revealed positivity for ANA, p-ANCA and anti-β2 glycoprotein. The proband's 1 year-old brother also possess same variant but is asymptomatic and without developmental concerns to date. The *IFIH1* mutation was not found in the proband's father or elder sister.

Like other mutations associated with AGS, gain-of-function mutations in *IFIH1* are associated with excess IFN-I production (7). To confirm the upregulation of IFN-I associated with the patient's *IFIH1* mutation, we performed transcriptome analysis of peripheral blood mononuclear cells by RNA sequencing. Compared to healthy controls, the patient exhibited global upregulation of IFN-I-inducible genes (Figure 2B). Despite the lack of clinical findings, the patient's brother and mother also displayed an interferon signature, albeit milder compared to the proband. These findings support the pathogenicity of the patient's *IF1H1* mutation in driving aberrant IFN-I production.

**Figure 3:**
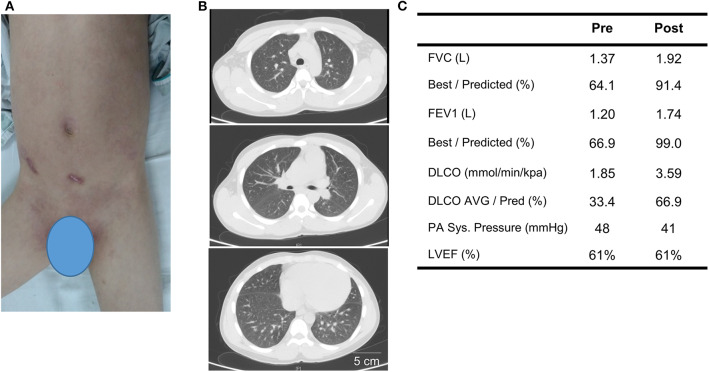
Treatment response to corticosteroids, cyclophosphamide, and tofacitinib after 3 months. **(A)** Post-treatment image of the skin illustrates resolution of generalized psoriasis in the abdomen and groin. **(B)** Repeat chest CT shows resolution of pleural effusion and improvement of ground-class opacities. **(C)** Comparison of key findings from pulmonary function test and echocardiogram pre- and 3 months post-treatment.

Before the genetic diagnosis was determined, the patient was started on immunosuppressive therapy for systemic autoimmunity with lung and renal involvement. His initial regiment consisted of methylprednisolone (1 mg/kg per day), thalidomide (50 mg PO daily) and IV cyclophosphamide (0.4 g weekly for 2 weeks, 0.4 g every 2 weeks for 6 weeks, then 0.6 g monthly for two additional months; cumulative dose = 3.2 g). Thyroxine tablets (20 mg PO daily) was given due the depressed thyroid function and irbesartan (150 mg daily) was started for hypertension and renal disease. With the availability of WES results after 1 month, the patient was started on the Janus kinase inhibitor (JAKinib) tofacitinib (titrated to 5 mg PO twice a day). Tofacitinib and other JAKinibs block signaling downstream of IFN receptor and their efficacy in AGS has been shown by a number of studies (9–12). Thalidomide was discontinued and oral prednisone was gradually tapered to 10 mg daily over the next 3 months. The patient showed significant improvement with treatment. The psoriatic plaques have mostly resolved (PGA-PsO score = 1) and features of ILD improved markedly on chest CT (Figures 3A,B; see Supplementary Figure 1 for side-by-side comparison of pre- and post-treatment images). Pulmonary function testing showed normalization of FEV1 and FVC, while DLCO and pulmonary hypertension (as measured by systolic PA pressure) also improved (Figure 3C; Supplementary Figure 1). Amelioration of proteinuria, hypocomplementemia, and hypothyroidism was also observed with treatment (Table 1). However, the patient's muscle weakness and difficulty with ambulation did not change significantly after treatment.

## Discussion

Gain-of-function mutations in *IF1H1* were first found to be responsible for a subset of AGS cases in 2014 (6, 7). The initial descriptions of early-onset encephalopathy, neurologic decline, autoimmune features, and presence of the interferon signature were compatible with the clinical spectrum of AGS associated with other causative genes. The phenotypic spectrum was later expanded to include Singleton-Merten syndrome (SMS), which manifests with dental dysplasia, osteopenia, and calcification of the aorta and heart valves (13–15). To date, about 40 cases of AGS and SMS associated with *IFIH1* mutations have been described in the literature.

The combination of neurologic disease with brain calcification, autoimmune thyroid disease, and immune complex-mediated kidney disease in our patient raised suspicion for AGS. The early age of disease onset, rapid clinical deterioration and the profound features of autoimmunity in a young boy are also suggestive of a monogenic etiology. However, the patient did not have the typical chilblain rash and psoriasis is uncommon in AGS. Psoriasis was described in two families with prominent musculoskeletal manifestations associated with *IFIH1* mutations at residue 331 (T331I and T331R) (16). McLellan et al. recently described a young child with AGS and a psoriasiform rash in a perianal, truncal, and facial distribution (10). A gain-of-function mutation in *IFIH1* (G495R) was found and the rash was thought to be associated with the patient's lupus-like disease. However, images or biopsy of the rash in these cases are not available for comparison.

Excess IFN-I production may be the trigger of the skin pathology because therapeutic use of IFN-I for hepatitis is known to induce psoriasis (17), although to our knowledge psoriasis has not been described in other types of AGS. Alternatively, psoriasis may be a unique feature associated with gain-of-function *IF1H1* mutations. In line with this view, loss-of-function variants of *IFIH1* were identified as protective alleles for psoriasis and psoriatic arthritis (18, 19), perhaps explaining why gain-of-function mutations may increase the risk for developing psoriasis. The response of the patient's psoriasis to treatment was also remarkable and maybe due to the efficacy of JAKinib for both interferonopathy and psoriasis (20, 21).

To our knowledge, ILD has not been reported in AGS. In contrast, ILD is observed in the majority of patients with SAVI (STING-associated vasculopathy of Infancy), a distinct interferonopathy characterized by vasculitis rash, arthritis and ILD caused by mutations of *TMEM137*, which encodes stimulator of interferon genes (STING) (22). Why ILD is associated with SAVI but not other interferonopathies is not clear given the similar global upregulation of IFN-I across all of these conditions. Expression of STING by cells in the lung parenchyma (type II pneumocytes, bronchial cells, and alveolar macrophages) leading to IFN-I-induced lung injury has been proposed (22). In contrast, the central nervous systems manifestations of AGS are less commonly seen in the SAVI. Therefore, although AGS and SAV are grouped under the umbrella of interferonopathies, further work is needed to clarify the mechanism(s) responsible for the heterogeneity of clinical manifestations among these conditions. The systemic autoimmunity in our patient may represent the initial trigger of lung inflammation, which is then perpetuated by the enhanced IFN-I signaling. While the characteristics of our patient's ILD on CT scan resembled the radiographic findings of SAVI-associated lung disease, histologic comparison is not available as the patient's lung disease improved steadily with treatment and a lung biopsy was not pursued.

Pulmonary hypertension can be caused by multiple etiologies based on the WHO (World Health Organization) Classification (23). The mechanism leading to development of pulmonary hypertension in our patient is likely multifactorial. Idiopathic pulmonary arterial hypertension (WHO Group 1) may arise from the lupus-like connective tissue disease. Alternatively, pulmonary hypertension may be attributed to left ventricular dysfunction (Group 2) or interstitial lung disease (Group 3). Although the patient did have small pericardial and pleural effusion, the contribution of left ventricular dysfunction is likely limited with normal ejection fraction and the lack of valve disease. Discriminating the roles of autoimmune disease and ILD in this case is difficult as the patient had long-standing disease by the time of presentation.

Jakinibs are increasingly utilized to treat AGS and other interferonopathies because the JAK kinases JAK1 and TYK2 mediate signaling downstream of the IFN-I receptor complex. The JAK1/2 selective inhibitors ruxolitinib and baricitinib are often chosen due to their highest affinity for JAK1 (9–12). The JAK1/3 inhibitor tofacitinib has also shown efficacy in SAVI (20). Tofacitinib was chosen as other Jakinibs are not yet available in our region. Aggressive treatment was warranted due to the patient's multi-organ disease and progressive deterioration. The patient responded well to the combination of corticosteroids, cyclophosphamide and tofacitinib. His skin rash, lung disease, and renal disease all improved with treatment. The improvement of his pulmonary function parameters are in line with the experience in some SAVI patients treated with JAKinibs (24). However, the treatment regimen did not lead to significant improvement in the patient's neurologic impairment and muscle weakness.

Common adverse effects of JAK inhibitors include increased low-density lipoprotein, neutropenia, and transient transaminase elevation. Serious infections are rare but cases of herpes virus and BK virus reactivation has been reported (11, 25). Most of these data are based on studies in adults with rheumatoid arthritis and pediatric data are lacking. Recurrent respiratory infection has been reported in a SAVI patient treated with ruxolitinib (24). The combined immunosuppressive effects of tofacitinib with corticosteroids and cyclophosphamide were certainly a concern. Fortunately, our patient did not experience any serious infection during the treatment period. Cumulative toxicity of cyclophosphamide is also associated with the risk of malignancy and infertility. Cyclophosphamide was weaned after 4 months and his daily oral prednisone dose has been gradually reduced to 10 mg daily.

Incomplete penetrance is well-recognized in AGS and disease phenotype and severity may vary considerably among individuals with the same mutation (1, 7). The proband's mother and brother both possess the pathogenic *IFIH1* mutation and a mild interferon signature but neither has shown any symptoms. Additional genetic and environmental factors that contribute to disease onset remain to be determined.

Our case highlights phenotypic heterogeneity of AGS and describe psoriasis and ILD as part of the clinical spectrum associated with gain-of-function *IFIH1* mutations. Interestingly, a recent study revealed the presence of pulmonary hypertension in some patients with AGS, particularly in those with *IFIH1* mutations (5). ILD in AGS may be under-recognized as chest imaging and pulmonary function test are not routinely performed. It is notable that the features of ILD seen in our patient reversed after treatment. Improvement of pulmonary function parameters and resolution of opacities on chest CT were noted after 3 months. Therefore, early detection and timely initiation of treatment can likely prevent the development of chronic lung disease. In view of our case and the previous association with pulmonary hypertension, we advocate detailed cardiopulmonary evaluation and monitoring for patients with AGS, especially those with *IFIH1* mutations.

## Data Availability Statement

All datasets generated for this study are included in the article/Supplementary Material.

## Ethics Statement

The studies involving human participants were reviewed and approved by Institutional Review Board at Guangdong Second Provincial General Hospital. Written informed consent to participate in this study was provided by the participants' legal guardian/next of kin. Written informed consent was obtained from the individual(s), and minor(s)' legal guardian/next of kin, for the publication of any potentially identifiable images or data included in this article.

## Author Contributions

SZ, PL, QZ, and TL designed the study. SZ, JW, SW, QH, YH, YL, and QZ collected data and performed analyses. SZ and PL drafted the manuscript. QZ and TL supervised the study. All authors approved the final manuscript as submitted and agree to be accountable for all aspects of the work.

## Conflict of Interest

The authors declare that the research was conducted in the absence of any commercial or financial relationships that could be construed as a potential conflict of interest.
