# Supplementary figures and images for: Interstitial Lung Disease and Psoriasis in a Child With Aicardi-Goutières Syndrome
Source: Front Immunol. 2020 May 20;11:985. doi: 10.3389/fimmu.2020.00985 (PMC7251162; doi:10.3389/fimmu.2020.00985)

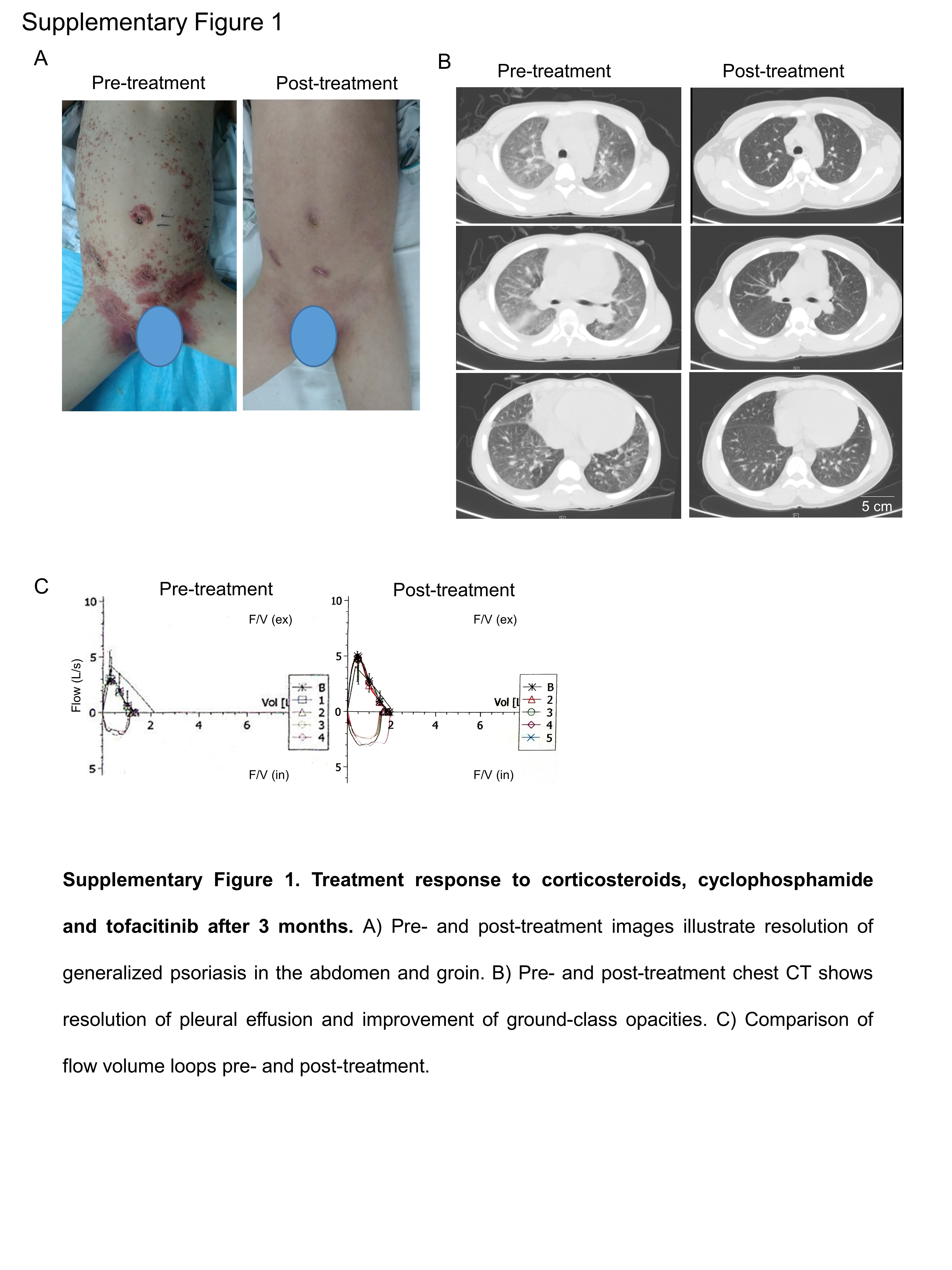

Supplement: Supplementary file 1 [file Image_1.jpg]
